# Letter to the Editor regarding "Lumbar facet joint denervation targeting the medial branch in the sub-mammillary fossa: An anatomical optimization study"^☆^

**DOI:** 10.1016/j.inpm.2025.100608

**Published:** 2025-06-28

**Authors:** Tomás Caroço, Eva Kubrova, Sahil Gupta, Mark Friedrich B. Hurdle

**Affiliations:** a Department of Physical Medicine and Rehabilitation, Hospital de Faro, Rua Leão Penedo, 8000-386, Faro, Portugal; b Algarve Biomedical Center Research Institute (ABC-RI), Faro, Portugal; c Department of Pain Medicine, Mayo Clinic, Jacksonville, FL, USA

Dear Editor,

We read with great interest the article *"Lumbar facet joint denervation targeting the medial branch in the sub-mammillary fossa: An anatomical optimization study,”* by John Tran et al. [1], which explores a promising new target for denervation of the lumbar facet joints.

Traditionally, facet joint denervation is performed by targeting the lateral neck of the superior articular process [2]. Anterolisthesis, characterized by an anteroposterior displacement of a vertebra [3], may consequently cause anterior displacement of the superior articular process's lateral neck. Importantly, the intervertebral foramina—through which the corresponding nerve roots exit—are anatomically defined by both the superior and inferior vertebrae. [4]. Thus, spondylolisthesis may result in narrowing of the intervertebral foramen, displacing the lateral neck of the superior articular process closer to the nerve root. This anatomical alteration increases the potential risk of proximity between the nerve root and the denervation target.

The authors propose that their novel sub-mammillary technique may be particularly beneficial in cases of severe scoliosis and/or spondylolisthesis. The objective of this letter is to present a clinical case involving mild anterolisthesis, in which the technique proposed by Tran et al. [1] might have offered increased safety.

## 1 Case presentation

We report the case of a patient who underwent lumbar medial branch radiofrequency ablation (RFA) at the L3–L4, L4–L5, and L5–S1 levels. The patient presented with grade I Meyerding classification L4 retrolisthesis (25 %) [5] and a left convex lumbar scoliosis (Cobb angle: 26.6°). The indication for RFA was based on two prior positive diagnostic medial branch blocks.

**Fig. 1:**
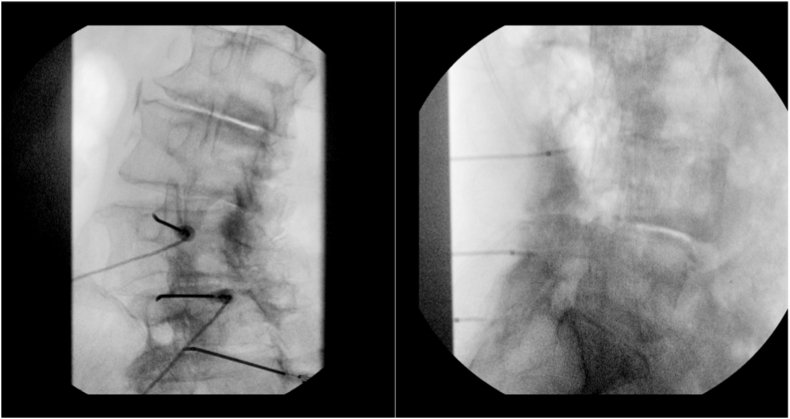
In the left image, the initial oblique view shows the cannulae in apparently correct positions. Following positive motor stimulation below the buttocks, the physician obtains a lateral view (right image), which reveals anterior displacement of the superior cannula.

The procedure began with cannula placement under fluoroscopic oblique view, targeting the lateral neck of the superior articular process (Fig. 1). However, motor stimulation at 2.0 mV elicited involuntary movement of the left leg. A lateral fluoroscopic view confirmed anterior displacement of the superior cannula into the foramen (Fig. 1). The needle was then repositioned posteriorly and inferiorly, with final confirmation under oblique view.

## 2 Discussion

Traditional targeting for lumbar facet denervation is challenging in cases of spondylolisthesis. Specifically, anteroposterior and oblique fluoroscopic views rely predominantly on the alignment of the inferior vertebra. In anterolisthesis, the displacement of the superior vertebra may decrease the distance between the cannula and the nerve root, increasing the procedure difficulty and risk. The target proposed by Tran et al. [1]—the superior portion of the sub-mammillary fossa, just inferior to the mammillary process—offers a potential safety advantage due to the presence of an anterior bony barrier that may prevent cannula proximity to the nerve root.

The identification of new anatomical targets for lumbar medial branch denervation is of significant clinical value. If validated, the approach proposed by Tran et al. [1] may improve the safety and efficacy of lumbar facet denervation, particularly in patients with spinal deformities.

## Declaration of competing interest

The authors declare that they have no known competing financial interests or personal relationships that could have appeared to influence the work reported in this paper.
